# Synthetic Peptides Induce Human Colorectal Cancer Cell Death via Proapoptotic Pathways

**DOI:** 10.1021/acsomega.4c08194

**Published:** 2024-10-11

**Authors:** Felipe P. Mesquita, Francisco L. de Oliveira, Emerson L. da Silva, Daiane M.S. Brito, Maria E.A. de Moraes, Pedro F.N. Souza, Raquel C. Montenegro

**Affiliations:** † Pharmacogenetics Laboratory, Drug Research and Development Center (NPDM), Federal University of Ceará, Fortaleza, CE 60430-275, Brazil; ‡ Cearense Foundation to Support Scientific and Technological Development, Fortaleza 60822-131, Brazil; § Red Latinoamericana de Implementación y Validación de guias clinicas Farmacogenomicas (RELIVAF), Madrid 28015, Spain

## Abstract

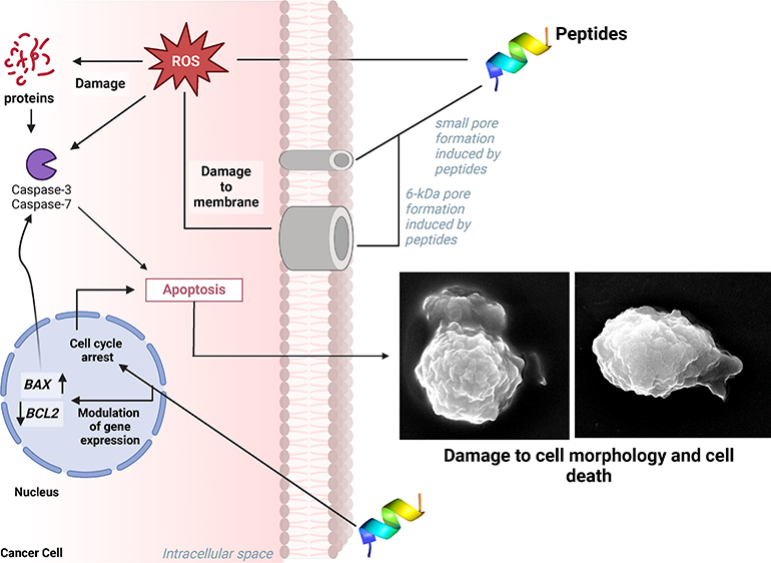

Cancer resistance to drugs and chemotherapy is a problem faced by public health systems worldwide. Repositioning antimicrobial peptides could be an efficient strategy to overcome that problem. This study aimed at repurposing antimicrobial peptides PepGAT and PepKAA for cancer treatment. After screening against several cancers, PepGAT and PepKAA presented IC50 values of 125.42 and 40.51 μM at 72 h toward colorectal cancer (CRC) cells. The mechanisms of action revealed that both peptides induced cell cycle arrest in G2/M and drove HCT-116 cells to death by triggering apoptosis. qPCR analysis revealed that peptides modulated gene expression in apoptosis, corroborating the data from caspase 3/7 and flow cytometry experiments. Yet, peptides induced ROS overaccumulation and increased membrane permeabilization, pore formation, and loss of internal content, leading to death. Additionally, peptides were able to inhibit cell invasion. Previous studies from the same group attested to no toxicity to normal human cells. Thus, PepGAT and PepKAA have great potential as anticancer molecules.

## Introduction

Cancer is a global problem, with an estimated 19.3 million new cases and being one of the leading causes of death worldwide, accounting for nearly 10 million deaths in 2020. The most common types of cancer worldwide are breast cancer, lung cancer, gastric cancer, leukemia, and colorectal cancer. Cancer can be treated in various ways depending on the severity of the patient’s condition and the stage of the cancer. Surgical resection is mostly employed for localized nonmetastatic stages depending on the location of the tumor and the stage of the cancer.^1−3^

Yet, chemotherapy is a rapidly growing method that utilizes drugs to interfere with the processes in cancer cells, such as their growth, development, dissemination, migration, and invasion, thus reducing the tumor or preventing cancer progression. This treatment can be carried out using a single drug or a combination of drugs, which is the most used approach due to its higher efficiency and more satisfactory response.^4−6^ Chemotherapy aims to stimulate cell death mechanisms, which can be triggered by direct or indirect DNA damage, resulting in dysregulation of tumor replication by genotoxic agents. These agents can be endogenous, such as reactive oxygen species (ROS), and exogenous, such as UV light, ionizing radiation (IR), and chemotherapeutic agents.^7,8^

The huge problem in cancer therapy is the development of resistance, limiting the cure in patients. Cancer can develop resistance virtually to all types of therapies, such as radiotherapy, chemotherapy, and even targeted therapies.^9^ Natural and adaptative resistance of cancer cells results from complex and collaborative events in the cellular response to drugs.^10^ Cancer resistance is classified as pharmacokinetic, which is the alteration of intracellular concentration of drugs, and pharmacodynamic, related to the failure to induce toxicity.^10^ This scenario urgently requires the development of new drugs to overcome resistance or even help the already available drugs become effective again.

Regarding alternative molecules, synthetic peptides (SAMPs) have already been studied and used as antimicrobial peptides due to their low toxicity levels and high antimicrobial activity.^11,12^ Here, two peptides, PepGAT (GATIRAVNSR, molecular mass 1044.90 Da) and PepKAA (KAANRIKYFQ, molecular mass 1239.00), were tested against cancer cell lines. Both peptides have an α-helix as a secondary structure and a hydrophobic ratio of 40%. PepGAT and PepKAA are cationic peptides with a positive net charge of +2 and +3, respectively.^11^ Recent studies have revealed that the mechanism of action of antimicrobial of PepGAT and PepKAA involves inducing the formation of pores in the cell membrane, thereby increasing intracellular concentration,^11−14^ which could also affect cancer cells. Toxicity studies showed that PepGAT and PepKAA were not toxic to rabbit and human (A, B, and O types) erythrocytes, fibroblasts from mice (cell line L929) and humans (cell line MRC-5), human keratinocytes (cell line HaCat), and embryo and larvae of zebrafish^11,15^ Therefore, this study explored how antimicrobial SAMPs could be repositioned to induce cytotoxicity in cancer cells and reveal insight into the mechanism of action behind anticancer activity.

## Methodology

### Ethical Statement

Not applicable to this study.

### Synthesis of Peptides

The synthetic peptides used in this study were bought from ChemPeptides company (http://chempeptide.com/) in Shanghai, China. After synthesis, the purity of peptides >98% was certified by high-performance liquid chromatography coupled with mass spectrometry analysis run by the company. For experiments, peptides were dissolved in DMSO 1% with 0.15 M NaCl in a laminar flow chamber to keep purity.

### Cell Culture

For AGP-01 (gastric adenocarcinoma), HCT-116 (colon cancer), MCF7 (cancer of breast), and SKMEL19 (malignant skin melanoma), the cell line was thawed and maintained with DMEM (Dulbecco’s modified Eagle’s medium) supplemented with 10% FBS (fetal bovine serum) and 1% antibiotics (penicillin/streptomycin 5000 U/5000 μg/mL). The cells were regularly maintained in a CO_2_ incubator at 37 °C and monitored using an inverted microscope (Leica DMIL LED). For maintenance or experimental manipulation, the cells were subjected to one wash with sterile PBS (Phosphate Buffer Solution) and detached using trypsin-EDTA 0.5% (0.25% v/v in 1× PBS). After dissociation, the trypsin-EDTA was inactivated, cells were counted, and experiments were carried out.

### Cytotoxicity by the Alamar Blue Assay

The Alamar Blue reagent was employed to evaluate the cytotoxicity effect of anticancer synthetic peptides against cancer cell lines. The 5-FU (fluorouracil) was used as positive control. First, the anticancer peptides were tested at a single concentration (100 μg/mL) against all cancer lines. Second, a curve concentration response for peptides (0–200 μg/mL) and 5-FU (0–40 nM). For both experiments, 3 × 10^3^ cells per well were seeded in a 96-well plate with a total treatment time of 72 h. Following the treatment, Alamar Blue was added at 0.2 mg/mL and incubated for 3 h. Finally, the fluorescent signal was read by a BioTek Synergy H1 (BioTek, Winooski, VT) plate reader at 560/590 nm excitation/emission.^16^

### Cell Cycle Analysis

In investigating the effects of peptides and 5-FU on the cell cycle, cells (3 × 10^4^ cells per well) were seeded in a 24-well plate and incubated for 24 h. Afterward, they were treated with 5-FU (23 nM) and peptides at IC50 for 24 h. Subsequently, the cells were harvested from all wells, collected, and centrifuged at 1500 rpm for 5 min. The pellet was then resuspended and fixed in 80% ethanol solution at 4 °C for 30 min to allow cell permeabilization. After centrifugation, the cells were resuspended and incubated with propidium iodide (50 μg/mL) for 25 min. The cells were then centrifuged again and resuspended in 1× PBS. A total of 10,000 events for each sample were analyzed by flow cytometry (BD FACSVerse). The FlowJo software was used to analyze the flow cytometry data.^17,18^

### Apoptosis Analysis by Flow Cytometry

Flow cytometry used propidium iodide (PI, 1 μM) to investigate the integrity of the cell membrane. HCT-116 cells (3 × 10^4^ cells per well) were seeded in a 24-well plate and incubated for 24 h with peptides and, afterward, for 24 h with the peptides (IC50) and 5-FU (IC50). The apoptosis induction in HCT-116 cells was performed by plating 3 × 10^4^ cells per well in a 12-well plate and treating for 24 h with the peptides (IC50) and 5-FU (IC50). After treatment, cells were washed with PBS, resuspended in 200 μL of annexin binding buffer consisting of 4 μL of annexin V-FITC, and further incubated with propidium iodide (PI) for 20 min (BD Pharmingen FITC Annexin V Apoptosis Detection Kit I). Afterward, they were resuspended in PBS, and a total of 10,000 events for each sample were analyzed by flow cytometry (BD FACSVerse).

### Invasion Assay

Cell viability was evaluated using the trypan blue exclusion assay.^19^ A total of 0.5 × 10^5^ cells were seeded in a 24-well plate and allowed to adhere for 24 h. Following this incubation period, 5-FU (23 μM), PepGAT (125.4 μM), and PepKAA (40.5 μM) were added to the cells. After an additional 24 h incubation, HCT-116 cells were collected and assessed for viability by counting live and dead cells in a hemocytometer chamber using a 0.4% trypan blue solution.

The invasion assay used Transwell inserts with 8 μm pore size and Matrigel coating in six-well plates (FALCON). Matrigel (20 μL) diluted in pure DMEM at a 1:6 ratio was added to the inside of each insert and incubated at 37 °C with 5% CO_2_ until the solution solidified. Next, 1 mL of serum-free DMEM with a concentration of 40 × 10^4^ HCT-116 cells was added to each insert and treated (upper chamber) with 5-FU (10 and 23 nM) and synthetic peptides at IC50. In the outside wells, 2 mL of DMEM with 10% FBS was added. After 24 h of incubation, the inner part of the inset was discarded, and the remaining cells that had invaded the matrix and reached the other side of the membrane were fixed and stained. The cells that crossed the pores and remained on the lower surface of the inset were stained with Giemsa dye and counted using an inverted microscope.^20^

### Fluorescence Microscopy Analysis

#### Cell Membrane Integrity by the PI Uptake Assay

It was also evaluated whether peptides could induce pore formation in the membranes of HCT-116 cancer cell lines by performing the PI uptake assay described by Oliveira et al.^12^ After the anticancer assay (under the same conditions described above), the samples were washed three times with 0.15 M NaCl (centrifuged at 5000*g* for 5 min at 4 °C), incubated with PI at 1 μM for 30 min at room temperature in the dark, and analyzed under a fluorescence microscope (Olympus System BX60) with an excitation wavelength of 488 nm and an emission wavelength of 525 nm.

#### Membrane Pore Formation by the FITC-Dextran Assay

In the same experiment, cells were also incubated with 10 μM of conjugated fluorescein isothiocyanate (FITC)-dextran with a size of 6 kDa (Sigma-Aldrich, São Paulo, SP, Brazil) for 30 min at 25 °C in the dark, washed the same as for PI, and analyzed and with an excitation wavelength of 490 nm and emission wavelength of 520 nm following the method described by Oliveira et al.^12^

### ROS Overproduction

To evaluate the peptide-induced ROS generation (H_2_O_2_), a fluorometric assay with DCFH-DA (2′,7′-dichlorofluorescein diacetate) was performed. After the anticancer assay, the samples were washed with 0.15 M NaCl (centrifuged at 5000*g* for 10 min at 4 °C). Next, 9 μL of DCFH-DA was added, and cells were incubated for 20 min at 22 ± 2 °C in the dark. Then, the samples were washed two times as described above and observed with a fluorescence microscope (Olympus System BX 41, Tokyo, Japan) with an excitation wavelength of 535 nm and an emission wavelength of 617 nm.^21^

### Apoptosis Induction by Caspase 3/7 Assay

The caspase activity was measured after cell incubation for 24 h, in the presence and absence of peptides, according to the methodology described by Souza et al.^15^ with some modifications. The cells were treated as above and then incubated using a 3 μL CellEvent reagent (ThermoFisher, São Paulo, SP, Brazil) for 30 min in the dark. Afterward, cells were washed and centrifuged as above. Finally, the cells were observed under a fluorescence microscope (Olympus System BX60) with an excitation wavelength of 342 nm and an emission wavelength of 441 nm.^22^

### Scanning Electron Microscopy (SEM)

The cancer cells untreated and treated with peptides were prepared and analyzed by SEM following Staniszewska et al.^23^ After the anticancer assay described previously, cells were fixed with 1% (v/v) glutaraldehyde in 0.15 M sodium phosphate buffer at pH 7.2 for 16 h. Next, the cells were washed three times with sodium phosphate buffer at pH 7.2 and centrifuged (5000*g* for 5 min at 4 °C) each time. Then, samples were dehydrated with increased ethanol concentrations (30, 50, 70, 100 and 100% [v/v]) for 10 min each at 25 °C and centrifuged as above each time. The final dehydration was performed with 50% (v/v) hexamethyldisilane (HDMS, Sigma, St. Louis, MI, USA) diluted in ethanol for 10 min, centrifuged as above, and then dehydrated with 100% HDMS. The dried cells were placed into a covered glass with a gold cloud using a coating machine (Emitech-Q150TES, Quorum Technologies, Lewes, England) and positron-emission tomography (PET). SEM analyses were run in a scanning electron microscope (Quanta 450 FEG, FEI, Waltham, MA) with a magnification of 20,000×.

### Total mRNA Extraction and qRT-PCR Analysis

For gene expression analysis, the HCT-116 cell line was plated in the concentration of 1 × 10^6^ cells/well in a six-well plate. After adherence, cells were treated with PepGAT and PepKAA for a noncytotoxic time of 24 h. After exposure, cells were trypsinized and collected, and the total mRNA extraction was performed by TRIzol Reagent (Life Technologies, USA) according to the manufacturer’s protocol. Then, the RNA concentration was determined using NanoDrop (Thermo Scientific), and reverse transcription was performed using the High-Capacity cDNA kit (Life Technologies, USA).

Quantitative real-time PCR (RT-qPCR) was performed with the Fast SYBR Green kit (Applied Biosystems, USA). Expression levels of *BCL-2* (NM_000633.3), *BAX* (NM_138764.5), *TP53* (*NM_*205264.1), *PARP1* (NM_001618.3), and *KRAS* (*NM_001369787.1*) were measured, and the relative expression levels were normalized and determined using *RPLP0* (NM_001101.5) gene as the reference for expression normalization between groups. Primer efficiency was determined for all of the genes described.

All requirements proposed in Minimum Information for Publication of Quantitative Real-Time PCR Experiments-MIQE Guidelines were followed.^24^ The expression level was calculated using the 2^–ΔΔCT^ method,^25^ considering nontreated cells (negative control) as a calibrator of the experiments.

### Statistical Analysis

The experiments were performed with three biological replicates and three technical replicates for each experiment. All data are shown as a mean ± standard deviation. In the in vitro experiments, analysis of variance (ANOVA) with Bonferroni’s post-test or Student’s *t* test was employed to compare the negative control (0.1% DMSO) with the treated groups. The Mann–Whitney or Kruskal–Wallis test was used to compare normal (healthy) gastric tissue samples in the gene expression with clinical specimens. Significant differences were considered with an interval of 95% (*p* < 0.05). GraphPad Prism 5.01 was used for data analysis and graph design.

## Results

### Synthetic Peptides Display Cytotoxic Activity against the Colorectal Cancer Cell Line

**Figure 1:**
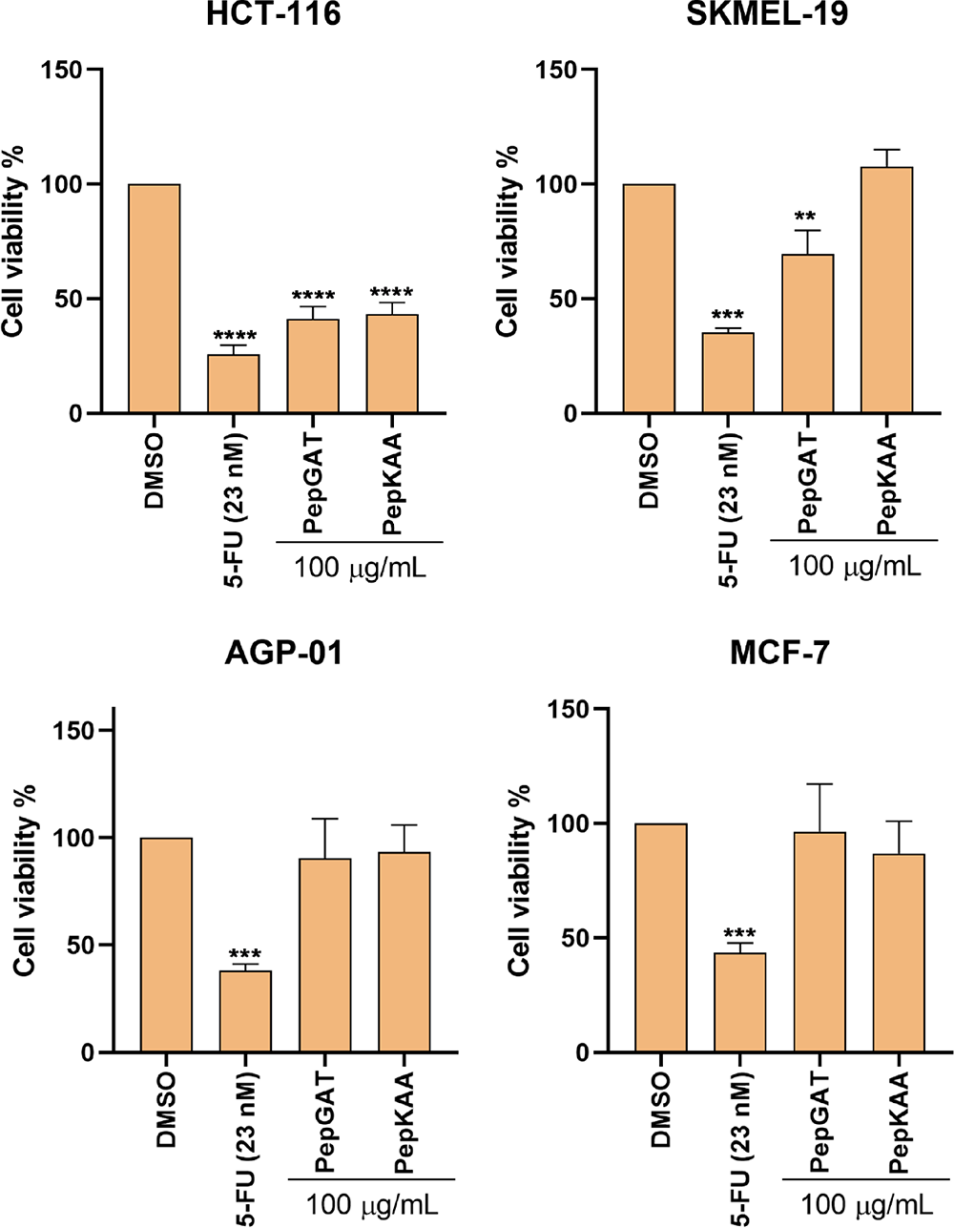
A cell viability assay was performed to evaluate the viability against five cell lines, including four cancer cell lines, i.e., AGP01, HCT-116, MCF7, and SKMEL19, treated with 5-FU (23 μM) and PepGAT at 95 μM and PepKAA at 81 μM (both at 100 μg mL^–1^) for 72 h. Data are represented as mean ± standard deviation of three independent experiments. Groups were compared to the negative control (DMSO) using ANOVA and Tukey’s post-test. Significant differences: ** *P* < 0.01, *** *P* < 0.001, and **** *P* < 0.0001.

The synthetic peptides PepGAT and PepKAA, at a concentration of 100 μg mL^–1^, were tested against four cell lines: AGP-01 (gastric adenocarcinoma), HCT-116 (colon cancer), MCF7 (cancer of breast), and SKMEL19 (malignant skin melanoma; Figure 1). The positive control for cytotoxicity to cancer cells was 5-fluorouracil (5-FU, 23 nM), and the nontoxic control was 0.1% of DMSO (Figure 1). Given the fact that the concentration of 5-FU is expressed in M, for comparison reasons, the concentration of peptides was expressed in the same unit of 5-FU. Therefore, 100 μg mL^–1^ corresponded to 95 μM for PepGAT and 81 μM for PepKAA. Among the cancer cell lines tested, the most affected was HCT-116, with an inhibition of 58.69% for PepGAT (95 μM) and 56.85% for PepKAA (81 μM) (Figure 1).

Due to the results against HCT-116, the potency efficacy was determined by employing a serial dilution curve (Table 1). The results revealed an IC50 of 23 nM, 125.42 μM, and 40.51 μM, respectively, for 5-FU, PepGAT, and PepKAA against HCT-116 (Table 1).

**Table 1:** Cytotoxicity of 5-FU Alone and in Combination with Synthetic Peptides against the Colorectal Cancer Cell Line (HCT-116)^a^

**compound**	**IC50**	**95% CI**
PepGAT	125.42 μM	82.9–257.1
PepKAA	40.51 μM	43.3–59.5
5-FU	23.3 nM	14.5–37.1

a 95 % CI: 95% confidence interval, IC50: half-maximal inhibitory concentration.

### Induction of Cell Cycle Arrest on HCT-116 Cells by PepGAT and PepKAA

The mechanisms of action were assessed to shed light on the events behind the anti-HCT-116 effect of the peptides. Before going through the mechanisms of action, it is important to clearly state that, although the IC50 was reached at 72 h, the mechanisms of action were assessed at 24 h. The aim is to understand the early (24 h) events induced by peptides that drove HCT-116 cells to death at 72 h. Studying the mechanisms of action at 24 h and the cytotoxicity (IC50) at 72 h can provide valuable insights into the effectiveness and behavior of peptides over time, in addition to understanding when the events that led cells to death at 72 h started.^26−31^

**Figure 2:**
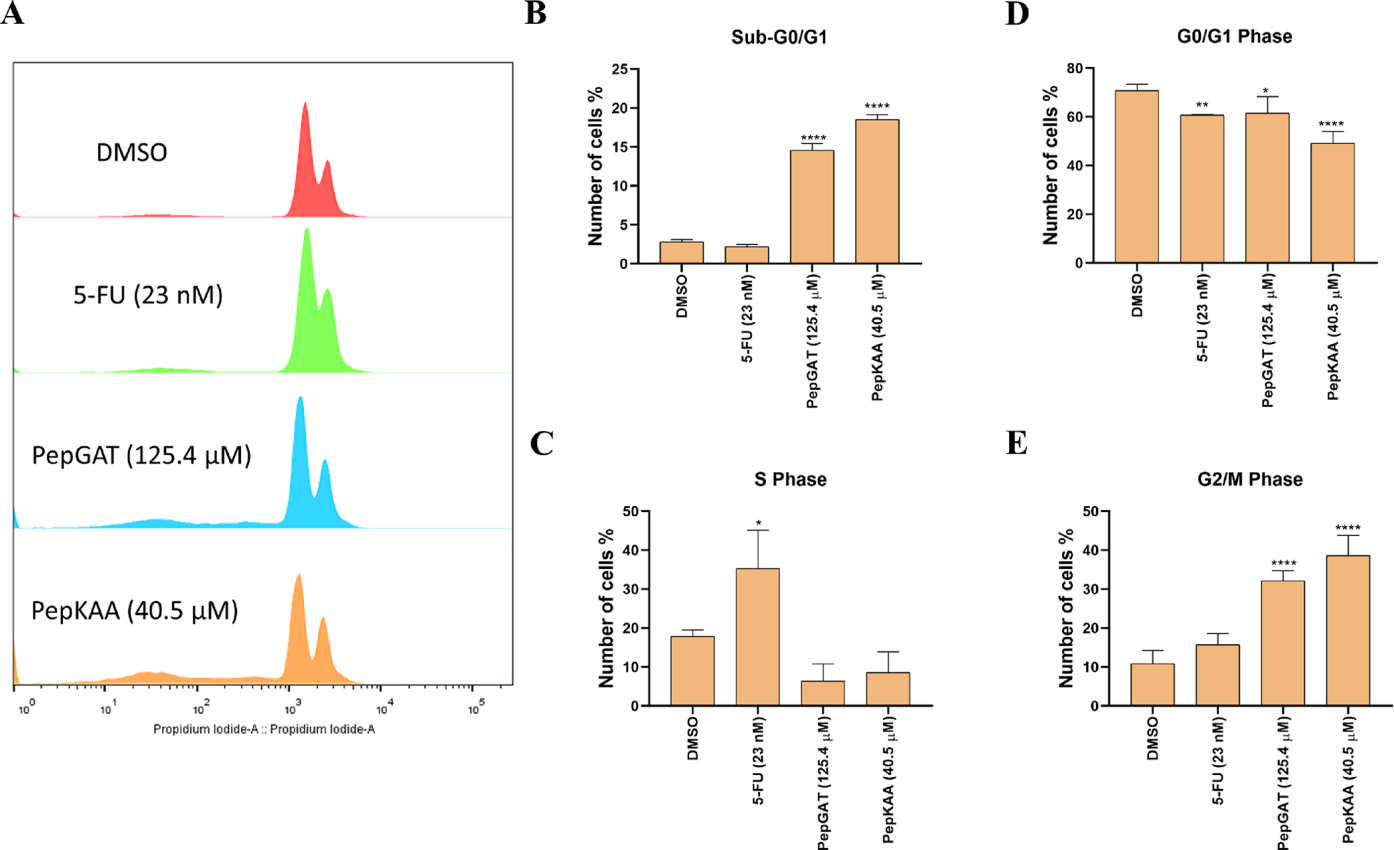
(A) Cell cycle distribution of HCT-116 cells after treatment with PepGAT and PepKAA at IC50 and 5-FU at IC50 concentration. The cell distribution in the (B) sub-G1, (C) G0/G1, (D) S, and (E) G2/M phases, calculated using FlowJo software, is represented as mean ± standard deviation of three independent experiments. Groups were compared to negative control (DMSO) using ANOVA and Tukey’s post-test. Significant differences: *P < 0.05, **P < 0.01, ***P < 0.001, and ****P < 0.0001.

First, it was evaluated whether peptides could interfere with the cell cycle of HCT-116 cells. As expected, DMSO did not affect the cell cycle of HCT-116 cells, and 25 nM 5-FU drove cells to cell cycle arrest in the S phase (Figure 2A). The peptides, however, did affect the cell cycle of HCT-116 cells, leading to a higher number of HCT-116 cells in Sub-G1, indicating the fragmentation of the nucleus (Figure 2B), reduction in the number of cells in the S phase (Figure 2C), and cell cycle arrest in the G2/M phase (Figure 2E).

### PepGAT and PepKAA Induce Caspases 3 and 7 and Activate the Apoptosis Pathway in HCT-116 by Promoting ROS Overproduction

**Figure 3:**
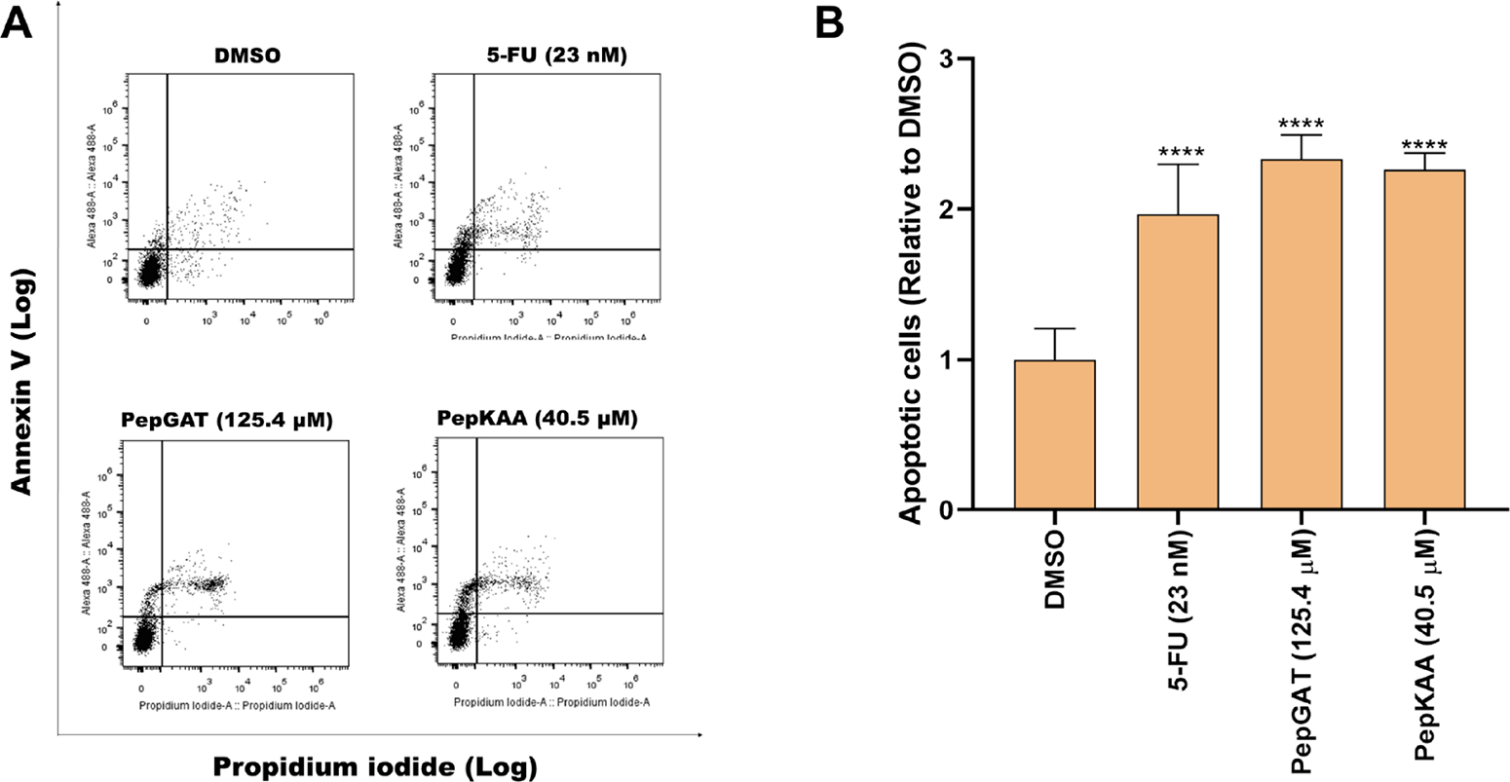
Cell death determination of HCT-116 cells after treatment with PepGAT at 125.4 μM, PepKAA at 40.5 μM (both at IC50), and 5-FU at 23 nM. (A) Flow cytometry histogram and (B) number of apoptotic cells in treatments. Data are represented as mean ± standard deviation of three independent experiments. Groups were compared to the negative control (DMSO) using ANOVA and Tukey’s post-test. Significant differences: *P < 0.05, **P < 0.01, ***P < 0.001, and ****P < 0.0001.

Flow cytometry was performed using PI and Annexin V assays to verify whether the peptides promote cell death after treatment (Figure 3A). The results showed an increase in apoptotic cells (PI+/Annexin V+) in the HCT-116 cells treated with the peptides compared to the control group (Figure 3B).

**Figure 4:**
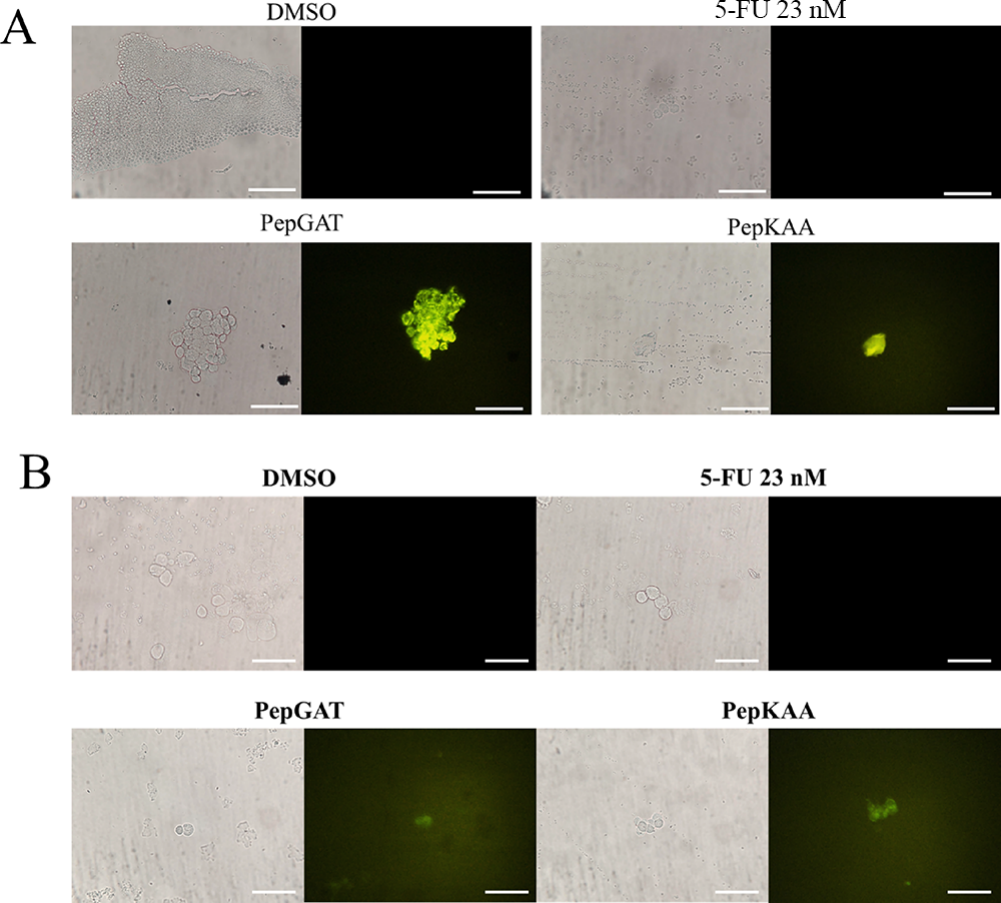
(A) Caspase 3/7-mediated (CellEvent Caspase-3/7 Detection Reagent) apoptosis and (B) ROS (DCFH-DA dye) overaccumulation in HCT-116 cells after treatment with PepGAT and PepKAA at IC50 and 5-FU (IC50). White bars indicate 100 μm.

The triggering of the apoptotic death pathway revealed by flow cytometry was confirmed by the activation of caspases 3 and 7 evaluated by fluorescence microscopy (Figure 4A). As revealed, DMSO or 23 nM 5-FU treated cells did not show any fluorescence, indicating no activation of caspases 3/7 on HCT-116 cells (Figure 4A). In contrast, PepGAT and PepKAA greatly induced apoptosis mediated by caspases 3/7 (Figure 4A). In the same context, it was analyzed whether peptides could induce ROS overaccumulation in HCT-116 cells, which works as a trigger for cell death. As expected, DMSO and 5-FU treated cells did not accumulate higher levels of ROS. However, in contrast, PepGAT and PepKAA induced ROS overaccumulation, as revealed by green fluorescence in treated cells (Figure 4B).

### PepGAT and PepKAA Induce Morphological Changes and Damage in the Membrane of HCT-116 Cells

**Figure 5:**
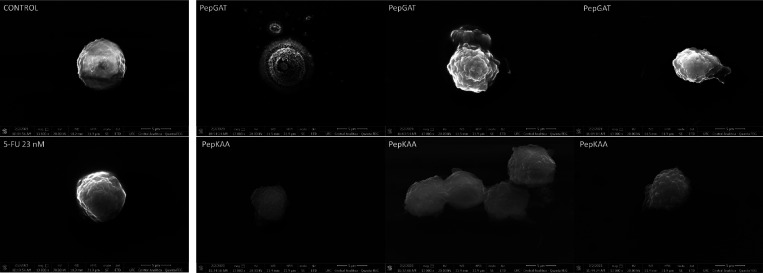
SEM analysis of HCT-116 cells after treatment with PepGAT and PepKAA at the IC50 and 5-FU at the IC50.

SEM analysis provided new information about how peptides induce morphological changes in HCT-116 cells (Figure 5). Control cells treated with DMSO (Figure 5, control panel) and 23 nM 5-FU (Figure 5 , 5-FU 23 nM panel) presented no damage to the cell morphology, spherical shape, normal cellular volume, or smooth surface. Otherwise, HCT-116 cells treated with peptides are completely damaged. PepGAT-treated HCT-116 cells presented severe and lethal damage to their morphology, such as alterations in cell shape, rough surface, cracks, cell deformation, and loss of internal content, indicating pore formation and membrane damage (Figure 5, PepGAT panels). Likewise, PepKAA-treated (Figure 5, PepKAA panels) cells presented alteration in the cell surface with increased roughness, cell deformation, and loss of internal content.

**Figure 6:**
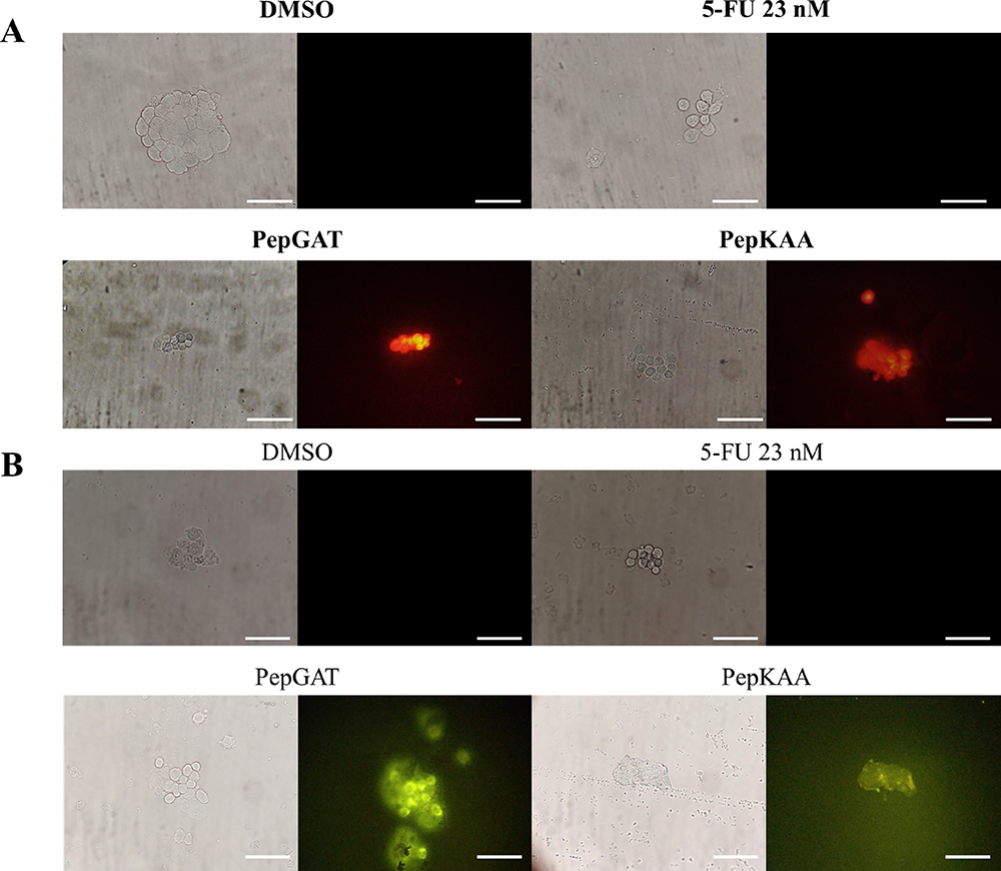
(A) Membrane permeabilization analysis by PI uptake assay and (B) membrane pore formation analysis by uptake of 6 kDa dextran-FITC in HCT-116 cells after treatment with PepGAT and PepKAA at IC50 and 5-FU (IC50). White bars indicate 100 μm.

The idea of pore formation presented by SEM analysis was further corroborated by serial fluorescence experiments in Figure 6. The PI uptake assay revealed that the treatment with peptides increased the membrane permeability of HCT-116 cells due to the red fluorescence in treated cells, which indicates PI’s movement through the membrane (Figure 6A). Moreover, the PI uptake assay by itself is just a suggestion of membrane permeabilization and is not necessarily indicative of pore formation. Therefore, a second experiment using dextran-FITC with a size of 6 kDa was performed. HCT-116 cells treated with peptides revealed a slight green fluorescence, indicating the movement of dextran-FITC through the membrane (Figure 6B). This result suggests that the pore formed has at least 6 kDa, allowing the accumulation of dextran-FITC within HCT-116 cells. The combination of peptides was also effective in inducing pore formation in HCT-116 cells (Figure 6B). However, both controls DMSO and 5-FU alone could increase the membrane permeabilization or induce pore formation.

### PepGAT and PepKAA Inhibit Invasion in HCT-116 Cell Lines

**Figure 7:**
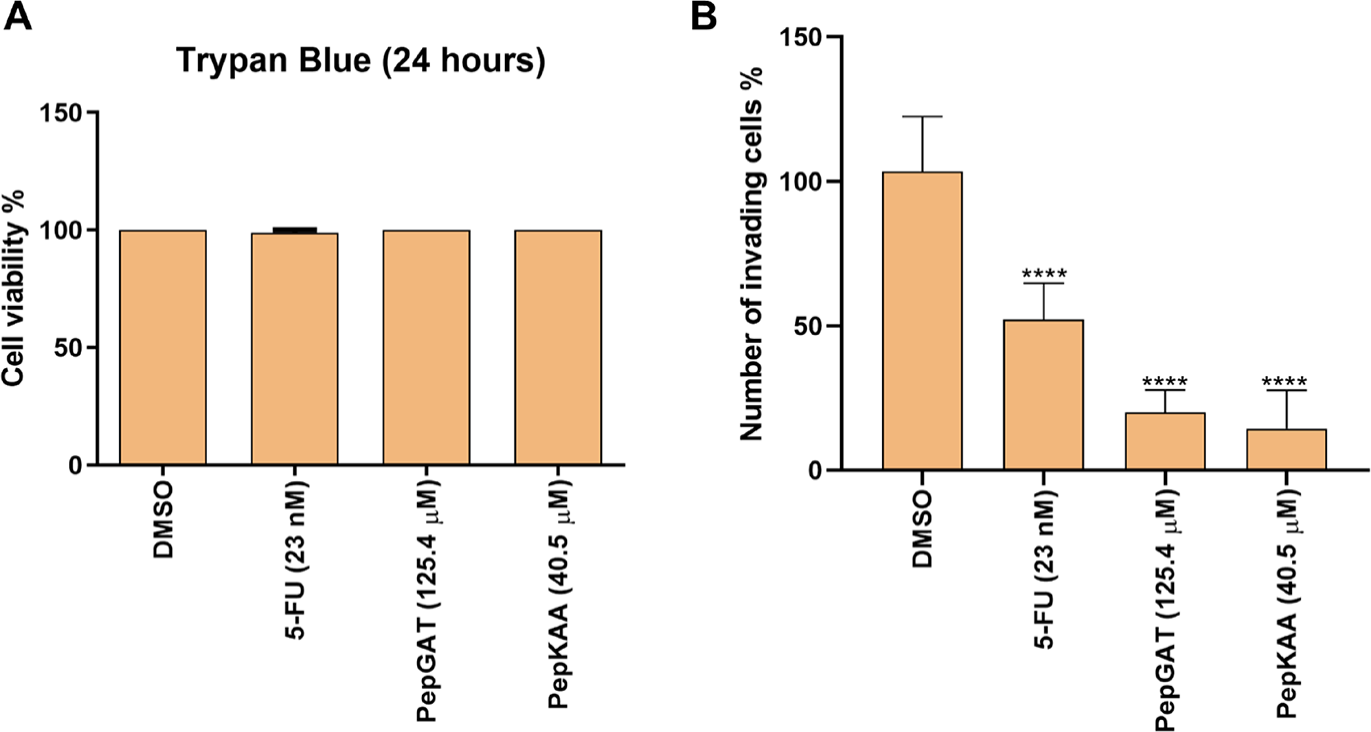
Cell invasion after treatment with PepGAT and PepKAA at IC50 and 5-FU (23 nM) for 24 h. (A) PepGAT, PepKAA, and 5-FU treatment did not affect cell viability after 24 h. Cell viability was tested by the trypan blue assay to demonstrate the noncytotoxic time and exclude the possibility of interference on invasion assay. (B) Number of invading cells performed using 0.8 μm diameter insert, allowing analysis at a single time point. Data are represented as mean ± standard deviation of three independent experiments. Groups were compared to negative control (DMSO) using ANOVA and Tukey’s post-test. Significant differences: ****P < 0.0001.

The cell invasion assay evaluated the ability of peptides to inhibit invasion in an extracellular matrix (Figure 7). The invasion assay was carried out in 24 h to avoid interference with the cytotoxicity effect of peptides. To test it, we performed a trypan blue assay in which cells treated with PepGAT (IC50) and PepKAA (IC50) with or without 5-FU did not show cytotoxic effects (Figure 7A). Regarding the invasion assay, HCT-116 cells treated with PepGAT and PepKAA presented a reduction of 70 and 75%, respectively, in the invasion process (Figure 7B). In the case of 5-FU, the inhibition of invasion was dependent on the concentration, with the best effective concentration being 23 nM, which inhibits in 50% the invasion of HCT-116 cells (Figure 7B).

### Synthetic Peptides Modulate Gene Expression in the HCT-116 Cell Line

In sequence, to better comprehend the intrinsic mechanism associated with the synthetic peptide treatment, the expression levels of pre- and proapoptotic transcripts *BCL2* and *BAX*, the tumoral suppressor gene *TP53*, the poly[ADP-ribose] polymerase 1 (*PARP1*) enzyme, and the *KRAS* oncogene were estimated by qRT-PCR after the treatment of HCT-116 with PepGAT and PepKAA for 24 h.

**Figure 8:**
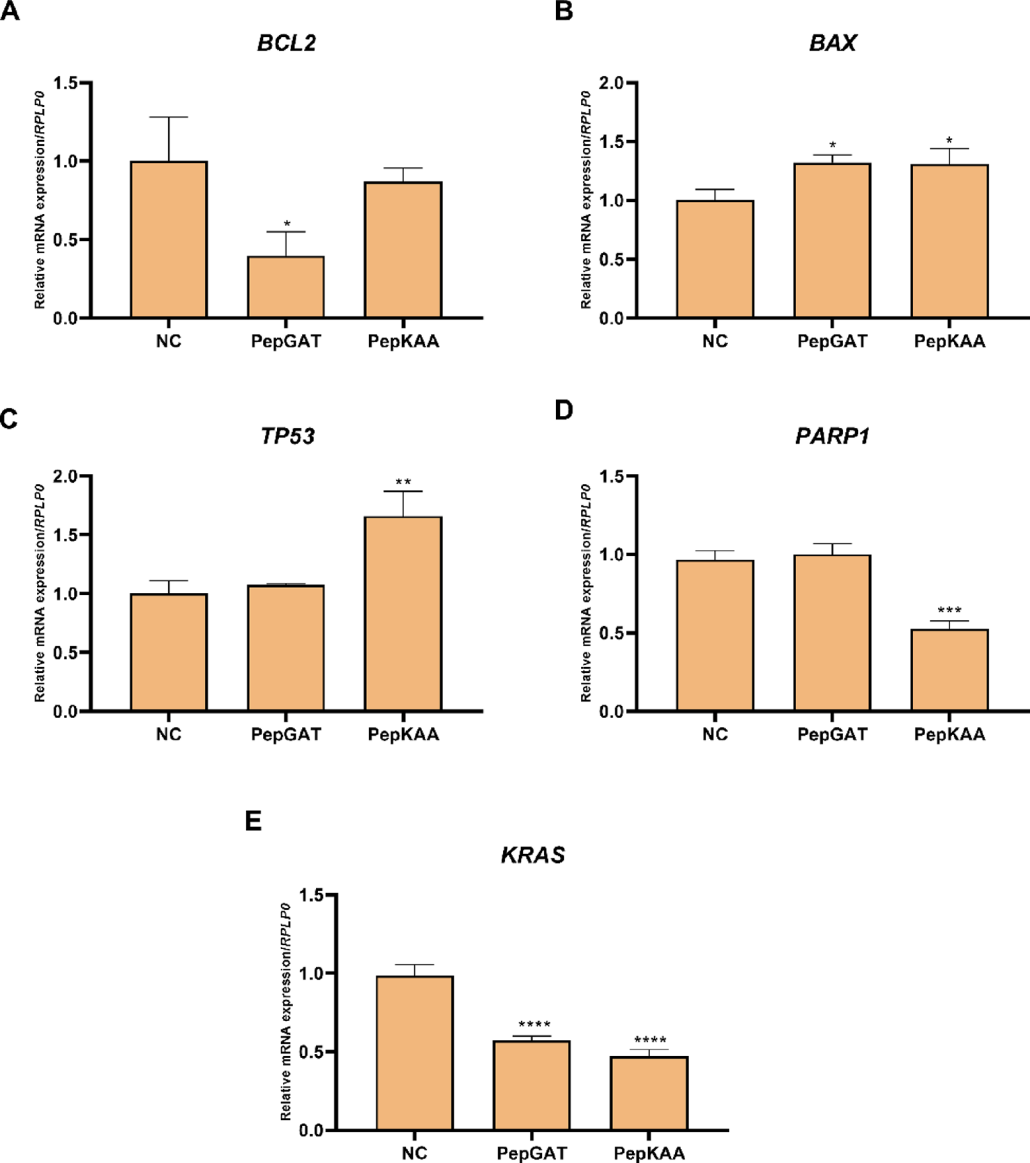
Synthetic peptides modify the expression levels of genes related to apoptotic pathways, DNA repair, and cellular growth and survival in the HCT-116 cell line. After 24 h of exposure to the synthetic peptides PepGAT and PepKAA, the mRNA levels of (A) *BCL2*, (B) *BAX*, (C) *TP53,* (D) *PARP1,* and (E) *KRAS* were analyzed by qPCR. RPLP0 levels normalized gene expression, and the NC group was used as a calibrator of the experiment. Data are presented as the mean ± SD of three independent experiments. Cells without previous treatment (NC) were compared to cells treated for 24 h with PepGAT and PepKAA with ANOVA followed by Bonferroni’s post-test. Significant differences: * *P* < 0.05, **P < 0.01, ***P < 0.001, and ****P < 0.0001. NC: negative control.

Data evidenced that PepGAT was associated with a reduction in *BCL2* (*p* < 0.05) and *KRAS* (*p* < 0.0001) and increased *BAX* (*p* < 0.05) mRNA levels. The mRNA levels of TP53 and PARP1 did not change in the presence of PepGAT (Figure 8). Concurrently, PepKAA led to a reduction in *PARP1* (*p* < 0.001) and *KRAS* (*p* < 0.0001) and increased the levels of *BAX* (*p* < 0.05) and TP53 (*p* < 0.01) (Figure 8). Altogether, these results bring new evidence into the mechanism of action of each synthetic peptide in the colon cancer cell line HCT-116. Even though they seem to have different intrinsic mechanisms, both led to the enhancement in the expression of the proapoptotic constituent *BAX* and reduction in the transcript levels of the *KRAS* oncogene (Figure 8).

## Discussion

Among the cancer types evaluated, colorectal cancer (CRC) was the most affected by the peptides tested. CRC had approximately 1.9 million new cases and ranked as the second most common cause of cancer-related deaths, with 576,858 deaths.^2^ In Brazil, it is estimated that there will be 704,000 new cancer cases per year between 2023 and 2025. In this case, CRC (46,000 new cases) is the second most common in males and females and the Northeast region, with an incidence rate of 10.99 per 100,000 individuals, indicating its importance.^32^ CRC refers to tumors found in the large intestine, specifically the colon and rectum, which are the final parts of the intestine before the anus.^32,33^

To combat CRC and slow its progression, the drug 5-FU is commonly used, especially as a first-line treatment for CRC.^34,35^ In this context, 5-fluorouracil (5-FU) is a uracil analog that contains a fluorine atom at its C-5 position instead of hydrogen, widely used in clinics and administered worldwide primarily for the treatment of CRC and other types of cancer as well.^36−38^ However, CRC becomes resistant to 5-FU treatment, and in some cases, surgery is the only way to cure patients.^38^

One of the mechanisms involved in 5-FU of CRC is increasing the mRNA levels of thymidylate synthase (TS), the target of 5-FU.^38^ CRC presented intrinsic higher levels of mRNA and accumulation of TS *in vitro*, *in vivo*, and in patients.^39^ This information has been used to direct new ways of treating CRC. The detection of higher levels of TS in colorectal tumors suggests that the patient should not be treated with 5-FU.^40^ On top of CRC resistance to 5-FU, the toxicity of 5-FU is another problem faced by patients. Although side effects go away at the end of the treatment, patients treating CRC with 5-FU experience side effects such as nausea, diarrhea, mouth sores, vomiting, loss of appetite, irritation and watery eyes, and low blood counts.^34,41^ Therefore, seeking and developing new molecules with potential against CRC and low toxicity to healthy cells and patients are urgently required. In this context, repositioning antimicrobial peptides (AMPs) against CRC has been an alternative approach researchers worldwide employ to combat the resistance of CRC to chemotherapy.^42,43^ However, due to low resistance to proteolysis and host cell toxicity, AMPs faced problems during clinical trials.^44^

Synthetic antimicrobial peptides (SAMPs) rationally designed for high activity and low toxicity have emerged as promising candidates to be repositioned for cancer treatment. Additionally, advancements in chemical synthesis have made synthetic peptides feasible for application.^11^ The SAMPs tested here, PepGAT and PepKAA, are multifunctional peptides with activity against bacteria, fungi, yeasts, and viruses.^11,14,15,22,45^

PepGAT and PepKAA possess two important characteristics that could facilitate their application. First, there is resistance to proteolysis. *In silico* and *in vitro* analysis revealed that PepGAT and PepKAA are resistant to digestion by enzymes present in the intestinal tract.^11^ Second, there is low toxicity to normal cells. *In vitro* analysis revealed that PepGAT and PepKAA presented no toxicity to human red blood cell types A, B, and O even in concentrations 10× higher than the antimicrobial concentration.^11^

Nevertheless, both peptides, like MRC-5, HaCat, and L92915, were not toxic to other human cells. *In vivo* experiments using zebrafish (*Danio rerio*) embryos and larvae revealed no toxicity of both peptides.^15^ Altogether, those results suggested that the peptides are not toxic and possess features for oral application, which would be useful for application against cancer.

Regarding the anticancer potential, PepGAT and PepKKA were more selective against HCT-116 cells, presenting IC50 values of 125.42 and 40.51 μM, respectively. This value is higher than IC50 of 23 nM presented by 5-FU (Figure 1 and Table 1). However, the comparison between concentrations for IC50 between peptides and 5-FU is not fair because they are from different chemical groups. Compared to that of other peptides, the anticancer activity revealed the potential of our peptides. For example, Hsu et al.^46^ evaluated the IC50 against colorectal cancer of Ple and Ple-a peptides. The Ple peptide did not reach an IC50 even with values higher than 500 μM; for Ple-a, the IC50 reached 197.3 μM. Won et al.^47^ reported that two synthetic peptides, GGN5 and V8W-GGN5^N11^, presented IC50 against HCT-116 cells at 193.31 and 144.37 μM. The authors also reported toxic potential against noncancerous cells.^46,47^

Compared with those peptides discussed, our peptides are required at a lower concentration to reach IC50. Altogether, based on these results, it is reasonable to suggest that PepGAT and PepKAA hold great potential to be explored as anticancer molecules. Based on that, the mechanisms of anticancer action were explored.

By evaluating the mechanisms of action, Hsu et al.^46^ revealed that Ple-a, at 197.3 μM, induced cell cycle arrest by increasing the number of cells in the sub-G1 phase. The results (Figure 2) of our study demonstrate a significant arrest of HCT-116 cells in the G2/M phase following treatment with the peptides PepGAT and PepKAA accompanied by an accumulation of cells in the Sub-G0/G1 phase, indicative of DNA damage and a precursor of apoptosis. This G2/M (Figure 2E) arrest suggests an interruption in cell cycle progression likely due to disruptions in the regulatory mechanisms. The cell cycle blockage in G2/M by interrupting the formation of the mitotic spindle is a common effect of drugs that disrupt the microtubules in cancer cells.^46,48^

The expression analysis of cells treated revealed a downregulation of BCL2 (Figure 8), an antiapoptotic gene, alongside an upregulation of the proapoptotic gene BAX (Figure 8), correlating with the increased apoptotic cell population in the sub-G0/G1 phase (although only about 15% of increase due to the 24 h treatment) (Figure 2B). Additionally, the elevated PARP1 expression, known for its role in DNA repair and apoptosis, further supports the induction of cell death by ROS overproduction in these cells,^49^ which also contributed to sub-GO/G1 accumulation. It is worth mentioning that 72 h treatment could not be possible to explain this mechanism because a great number of cells are dead. Interestingly, alterations in KRAS expression, which play a pivotal role in cell proliferation and are relevant colorectal cancer biomarkers, might also contribute to the observed effects, especially in G2/M arrest. A literature review listed several studies demonstrating KRAS regulation on cell cycle progression upon DNA damage.^50^

A study has shown that the inhibition of KRAS can lead to cell cycle arrest at the G2/M phase, primarily due to its role in DNA damage response (DDR) signaling. KRAS is known to activate pathways that influence cell cycle progression, including the G2/M checkpoint. When KRAS is blocked, this disrupts the proper activation of cyclin-dependent kinases and other regulatory proteins required for mitosis, leading to G2/M arrest. This mechanism highlights the potential therapeutic strategy of targeting KRAS to induce cell cycle arrest in cancer cells.^50^ These combined gene expression changes suggest that the therapeutic effect of PepGAT and PepKAA may be mediated through a synergistic mechanism involving cell cycle arrest and the activation of apoptotic pathways, presenting a potential strategy for targeted cancer therapy.

Cell cycle blockage in cancer cells leads to severe damage that cells cannot recover from. Cell cycle arrest in the G2/M phase indicates that the cancer cell loses control of its cycle and drives it to death by apoptosis.^51^ Followed by inducing a stop in the cell cycle in HCT-116, PepGAT and PepKAA increase 2-fold the number of apoptotic cells as revealed by the annexin assay (Figure 3), apoptosis mediated by caspase 3/7 (Figure 4A) and the overaccumulation of ROS (Figure 4B).

Figures 2–4 presented a sequence of events by somehow leading PepGAT and PepKAA to HCT-116 cells to death. Peptides induced an arrest in the cell cycle in G2/M (Figure 2), which induced cells to go into undesired pathways, leading to apoptotic events (Figure 3 and 4A) and the overaccumulation of ROS and, ultimately, cell death. As far as we know, this is the first study to show these sequential events ending in ROS accumulation in cancer cells. Additionally, it is important to note that the mechanisms presented by PepGAT and PepKAA differ from those presented by 5-FU.

It is important to discuss that the overaccumulation of ROS in HCT-116 cells is not necessarily the end of the road; otherwise, it could be the start. ROS is a natural byproduct of metabolism, requiring a tiny regulation to avoid accumulating higher levels in cells.^52,53^ It is known that tumor cells suffer more than normal cells due to high levels of ROS and can easily die from damage caused by them.^54−56^ The uncontrolled ROS level imbalance in cells led to damage to molecules such as DNA, proteins, and lipids, driving cells to death. Thus, it is feasible to hypothesize that the overaccumulation of ROS induced by PepGAT and PepKAA (Figure 4B) could act in two ways, driving DNA damage: (1) damage to important proteins or lipid peroxidation leading to apoptotic events and cell cycle arrest and death and (2) the higher levels of ROS could directly damage the DNA, interfering in the cell cycle and leading to apoptotic events and cell death.

High ROS levels could lead to the oxidation of proteins and lipids in cells, which, in turn, triggers lethal damage to cells.^54−56^ For example, Zhang et al.^57^ revealed by atomic force microscopy that high levels of ROS in prostate cancer cells induced by paclitaxel lead to the disruption of microtubules. Tubulin is important during the cell cycle because it commands the separation of chromosomes for new cells by forming the mitotic spindle.^54,58^ Damage to microtubules may result in problems during the cell cycle and cell death. Here, PepGAT and PepKAA induced a high accumulation of ROS in HCT-116 cells, cell cycle arrest in G2/M, and induction of apoptosis (Figures 2–4).

Additionally, higher levels of ROS induce membrane lipid peroxidation, altering membrane permeability, integrity, curvature, organization, and, hence, pore formation.^54^ The induction of pore formation on paclitaxel-prostate cancer cells is ROS-dependent.^57^ That is an interesting point because Aguiar et al.^22^ revealed that in *Cryptococcus neoformans* cells, PepGAT induced pore formation by a ROS-dependent pathway, and PepKAA induced pore formation by a ROS-independent pathway. Based on that, it is feasible to suggest the correlation between ROS overaccumulation and pore formation in HCT-116 cells (Figures 5 and 6).

It is worth mentioning that the peptides used in this study were proven cell-penetrating antimicrobial peptides^11,22,45^ driven to anticancer potential. Based on that, two questions arise: Could antimicrobial peptides be driven to attack the membrane of cancer cells? Are they selective for cancer cell membranes?

Regarding the first question, the short answer is yes. The long one requires an additional explanation. AMPs have been repositioned for cancer treatment due to similar characteristics shared by microorganisms and cancer cells, the negatively charged membrane.^59−61^ Nowadays, it has been largely discussed and accepted that cancer cells possess negatively charged membranes.^62−64^

The negative charge comes from the high phosphatidylserine concentration, heparin sulfate from proteoglycans, and the hypoxia environment.^65−67^ The negative charge of the membrane makes cancer cells susceptible to the cationic peptides (positively charged).^59−61^ Souza et al.^11^ reported that PepGAT and PepKAA possess a positive net charge of +2 and +3 due to arginine and lysine residues added during the design and discuss that this characteristic is important to peptides to attack microbial membranes, which are also negatively charged.

The answer to the second question is related to the first. The characteristics that made cancer a target for antimicrobial peptides are the same as those that exclude noncancer cells as a target. The membranes of noncancerous cells enclose the phosphatidylserine in the cytoplasmatic side of the membrane, leading to the outside layer of the noncancerous membrane with a neutral charge not attracting cationic peptides.^59−61^

Cell membranes have a complex system composed of lipids, proteins, and sterols that stabilize the membrane.^59−61^ These plasma membranes can undergo remodeling due to external stimuli, forming pores in the membrane. SAMPs can form pores in the membrane, although not all do it. By attacking the membrane, peptides challenge cancer cells to develop resistance.^11^ Over cellular evolution time, membranes retain the same conserved structure with slight variation. Therefore, inducing a change in the membrane is metabolically expensive aside being dangerous to the cell. It is an important mechanism against cellular resistance.^11^

Together, SEM and fluorescence microscopy revealed the ability of PepGAT and PepKAA to induce pore formation and the loss of internal content in HCT-116 cells (Figures 5 and 6). Despite being separated experiments, Figures 5 and 6 reveal the sequence of events. The PI uptake assay (Figure 4a) revealed that the membrane of peptide-treated HCT-116 cells had an increased membrane permeability. The PI uptake assay is not a definitive assay to indicate pore formation because it has a size of 0.1 nm in diameter, which means that membranes permeable to PI very likely hold pores with this size.

Etxaniz et al.^68^ (2018) revealed that some cells could recover from small pores that allow PI movement. However, dextran-FITC was used, revealing pores of at least 6 kDa (Figure 4b) in HCT-116 membranes, which can cause significant problems for cell function by allowing the movement of small molecules and other substances. Pores allowing the movement of dextran-FITC at least 1 nm in diameter are classified as large pores, leading to extravasation of cytoplasmic content, depolarization of membranes, and apoptosis.^68^ On top of that, SEM analysis corroborated the hypothesis of pore formation by revealing that HCT-116 cells treated with PepGAT and PepKAA are completely damaged with signals of loss of internal content.

Altogether, these effects are related to the ability of PepGAT and PepKAA to inhibit the invasion profile of the HCT-116 cells. It was clear that in the presence of peptides, the number of invading cells is dramatically reduced even more than in cells treated with 5-FU. The invasion ability of cancer cells is involved in establishing metastasis, a stage of cancer with a poor prognosis for the patient, which is directly linked to cellular invasion.^16−18,69^

## Conclusions

Our results revealed that antimicrobial peptides PepGAT and PepKAA possess an anticancer potential against CRC. Peptides exert their anticancer activity by multiple mechanisms of action, which could make it difficult for CRC to develop and acquire resistance. A previous study from our research group revealed that both peptides have no toxicity to human cells and zebrafish embryos. Altogether, the results suggest the potential of peptides as anticancer molecules to develop potential new drugs.

## Data Availability

No data sets were generated or analyzed during the current study.
